# Impact of video-led educational intervention on the uptake of influenza vaccine among adults aged 60 years and above in China: a study protocol for a randomized controlled trial

**DOI:** 10.1186/s12889-021-10220-1

**Published:** 2021-01-27

**Authors:** Pengchao Li, Khezar Hayat, Minghuan Jiang, Zhaojing Pu, Xuelin Yao, Yamin Zou, Krizzia Lambojon, Yifan Huang, Jinghua Hua, Hanri Xiao, Fulei Du, Li Shi, Panpan Zhai, Wenjing Ji, Zhitong Feng, Yilin Gong, Yu Fang

**Affiliations:** 1 Department of Pharmacy Administration and Clinical Pharmacy, School of Pharmacy, Xi’an Jiaotong University, Xi’an, 710061 China; 2 Center for Drug Safety and Policy Research, Xi’an Jiaotong University, Xi’an, 710061 China; 3 Shaanxi Centre for Health Reform and Development Research, Xi’an, 710061 China; 4 Research Institute for Drug Safety and Monitoring, Institute of Pharmaceutical Science and Technology, China’s Western Technological Innovation Harbor, Xi’an, 710061 China; 5 Institute of Pharmaceutical Sciences, University of Veterinary and Animal Sciences, Lahore, 54000 Pakistan; 6 Department of Pharmacy, the Hospital of Xi’an Jiaotong University, Xi’an, 710049 China

**Keywords:** Intervention, Older adults, Influenza vaccine, Knowledge, Practice

## Abstract

**Background:**

Influenza is a global health threat to older adults, and the influenza vaccine is the most effective approach to prevent influenza infection. However, influenza vaccination coverage among Chinese older adults is far less than in developed countries such as the United States (4.0% vs. 64.9%). This study aims to increase influenza vaccination coverage in Chinese adults ≥60 years using a video-led educational intervention conducted by medical students.

**Methods:**

A cluster randomized controlled trial will be conducted in 4 districts of Xi’an city, Shaanxi Province, China, using a stratified sampling approach. Adults aged ≥60 years will be recruited from 8 community hospitals. A self-administered questionnaire of knowledge, attitudes, and practices (KAP) will be employed to record the KAP score. During the 6-month interventional period, participants in the intervention group will receive educational videos focused on influenza and influenza vaccination, coupled with a group discussion conducted by the medical students. For those in the control group, no intervention will be provided. The outcomes measured in both groups will be the influenza vaccination coverage and the KAP scores of all participants.

**Discussion:**

Medical students are more likely to educate older adults about scientific knowledge of influenza and its vaccine compared to clinical practitioners, who, most of the time, remain over-occupied due to the extensive workload. Video-led counseling and education could be a useful option to optimize older adults’ understanding of influenza and influenza vaccination. This eventually could improve the uptake of influenza vaccine among Chinese older adults.

**Trial registration:**

Chinese Clinical Trial Registry; ChiCTR2000034330; Registered 3rd July 2019.

**Supplementary Information:**

The online version contains supplementary material available at 10.1186/s12889-021-10220-1.

## Background

Influenza, an infectious disease caused by the influenza virus, can threaten human health, especially during an influenza pandemic [1]. Every year, 291,243 to 645,832 people die due to seasonal influenza [2]. Influenza related risk of death is higher in older people, such as 84–95% of influenza-associated deaths occur in older adults [3–6].

Influenza vaccination, which is the most effective measure to prevent influenza, can effectively reduce the rate of hospitalization and deaths owing to influenza-associated severe acute respiratory infection [7–9]. Besides, it may produce indirect herd protection for the unvaccinated population in communities [10]. Numerous studies have illustrated influenza vaccination as a cost-effective strategy from the societal perspective to limit influenza progression [11, 12].

In 2019, 18.1% of the total population of China (0.254 billion people) were adults aged 60 years and above [13]. The guidelines for Seasonal Influenza Vaccination in China (2019–2020) [14] recommend that older adults should be prioritized to vaccinate. Nevertheless, the coverage of the influenza vaccine in Chinese older adults remains low (4.0%) than in the United States (64.9%) [15, 16].

Inadequate knowledge, lack of confidence, and complacency (underestimated risk of disease or risk denialism) [17] could be significant reasons for low influenza vaccine coverage among older people in China. A study [18] showed that only 7 and 4% of older adults knew about the influenza virus and vaccine, respectively. In the past, the poor quality of vaccines caused life-threatening effects that lead to several deaths. It diminished the level of confidence about the safety of vaccines among the public [19, 20]. A previous study [18] indicated that more than 55% of the older adults thought they could not get seriously ill from influenza.

The Healthy China Initiative (2019–2030) has emphasized that people should recognize the critical role of vaccines in disease prevention. High-risk groups, including older adults, should get vaccinated before the flu season [21]. Thus, older adults must improve their knowledge, which will increase the uptake of vaccines in the future. Several studies have indicated that educational intervention regarding influenza and its vaccine is one of the practical approaches to enhance community knowledge and confidence [22].

A recent Chinese survey reported that the educational level of two-thirds of older adults is primary school or below [23], which could track to low health literacy, as found in a previous study [24]. Therefore, it may not be suitable to educate older adults through brochures and text messages due to poor health literacy. Still, video-led counseling could be more productive and fruitful in this age group [25]. Medical students, who have relatively abundant knowledge about infectious diseases compared to the public, can communicate with older adults effectively [26]. There are more than three million medical students at different medical schools in China [27]. They have a more flexible time to conduct health education about influenza and influenza vaccines for older people than a clinical practitioner.

This study aims at improving knowledge on influenza and increasing the uptake of influenza vaccination in older adults using a video-led intervention coupled with a group discussion conducted by medical students.

## Methods

### Study design

This is a CONSORT-compliant cluster randomized controlled trial that will determine the effectiveness of video-led counseling to older adults about influenza and influenza vaccination by medical students [28]. Stratified cluster-based sampling will be employed in this study. Based on the socioeconomic status of every district in Xi’an city, Shaanxi Province, four districts will be selected out of 13, including two districts that have high socioeconomic status, including Xincheng and Yanta, and two districts that have low socioeconomic status, including Chang’an and Baqiao. Two community hospitals will be chosen randomly from each district using computer-generated random numbers. In total, 8 community hospitals will participate in this trial (4 hospitals of intervention and 4 hospitals of the control group). The older adults will be chosen randomly by the health management system of Xi’an city. The Chinese guidelines recommend vaccination should preferably be completed by the end of October. If people do not receive the vaccine at that time, vaccination will be available throughout the flu season. Therefore, the intervention period (six months) will have two stages. One is before the flu season (August, September, October), and one is during the flu season (November, December, January).

### Study participants

Eligible old residents attending this program will be screened with the help of the principal investigator and nurses in community hospitals. Inclusion criteria include 1) participants aged 60 years and above; 2) living in the community for the last one year; 3) people willing to participate and provide written informed consent. Exclusion criteria include 1) Those who have cognitive impairment and cannot cooperate; 2) People with serious mental illness. Additionally, those below 60 years of age and who are unwilling to finish the questionnaire will be excluded. A six-item screener will be used to measure the cognitive impairment of potential study participants as it is brief, reliable, and easy to administer [29, 30]. Besides, auditory and visual assessments of the participants will also be conducted.

### Sample size

According to previous studies [31], the influenza vaccine coverage among older adults was almost 2% in western China. The relative risk of intervention ranged from 1.04 to 6.22 in the uptake rate compared to the control group. We hypothesized that the influenza vaccine’s uptake rate would increase from 2 to 10% by employing a medical student-led educational intervention. A mathematical formula [32] was used to calculate the minimum sample size. Eighty participants will be required for each group with a study power of 0.90 and a significance level of 0.05. Considering the loss of follow-up and the low response rate, the sample size will be 200 participants for one group.

### Video design

A video will be designed and made by a professional company under the supervision of three experts from the China Centers for Disease Control and Prevention (CDC) and university hospitals. They will help us check the content of the video. A pilot test will be performed to determine the understanding of older adults. Eventually, the video content will consist of 1) influenza knowledge, which covers symptoms, complications, transmission routes, treatment measures, and prevention. 2) influenza vaccine knowledge, including types of vaccine, time of injection, health benefits, and 3) influenza pandemic cases globally.

### Data collection instrument

A questionnaire-based on the Health Belief Model (HBM) has been developed after a literature survey [18, 33–37]. A panel comprising three experts on public health and ten older adults established the content and face validity of the questionnaire. Thirty older adults were recruited in the pilot testing, and the questionnaire was administered over the telephone by medical students. The reliability of the final form of the questionnaire was assessed by measuring the value of Cronbach’s alpha, which was excellent (Cronbach’s alpha = 0.844).

The questionnaire consisted of four sections, including basic sociodemographic characteristics, knowledge part, attitude part, and behavior part (supplementary file). Gender, age, occupation, education, monthly income, and chronic disease were listed in the sociodemographic part. There were 10 true or false questions regarding influenza and influenza vaccine in the knowledge part with yes, no, and unclear options. The knowledge score will be calculated using correct answers with a maximum score of 10 points. Moreover, the knowledge of the participants will be categorized into three classes, such as poor (< 4 points), average (4–7 points), and good (> 7 points).

Eight questions were asked to explore the attitude about the risk of influenza infection, the burden of disease, safety, effectiveness, and treatment. Here, the Likert scale was used to get an attitude score. Hence, 40 points are as full scores (strongly agree-1 point, agree-2 points, uncertain-3 points, disagree-4 points, and strongly disagree-5 points).

Six practice-related questions were designed to determine the behavior of prevention about influenza treatment in influenza-like illness (ILI), willingness to accept the vaccine. The practice score, a maximum of 12 points, will be calculated based on the response of participants (yes-1 point, no-2 points).

### Intervention procedures

The medical students will be recruited from high grades in medical colleges. Screening criteria include 1) those who are well versed in information about various diseases and vaccination; 2) those who could have a better understanding of our study protocol; 3) those who like to educate older adults. Before the formal investigation, medical students will be provided four hours of focused training to increase their understanding of influenza, influenza vaccination, questionnaire items, and video-related professional knowledge. Additionally, a session of training will also be conducted to improve their communication skills, which will help them to better communicate with older adults. The training adequacy of medical students will be conducted through group discussions during which they will be asked to comment directly on their readiness to play their defined role. Any component of the training that will be considered insufficient would subsequently be addressed.

Before the intervention, medical students will call the older adults, enquiring about their willingness to participate in this program. After their verbal agreement, older people in the control and intervention group will fill the questionnaires under the supervision of a medical student. Their knowledge, willingness to accept the vaccine, and uptake rate will be assessed.

**Fig. 1:**
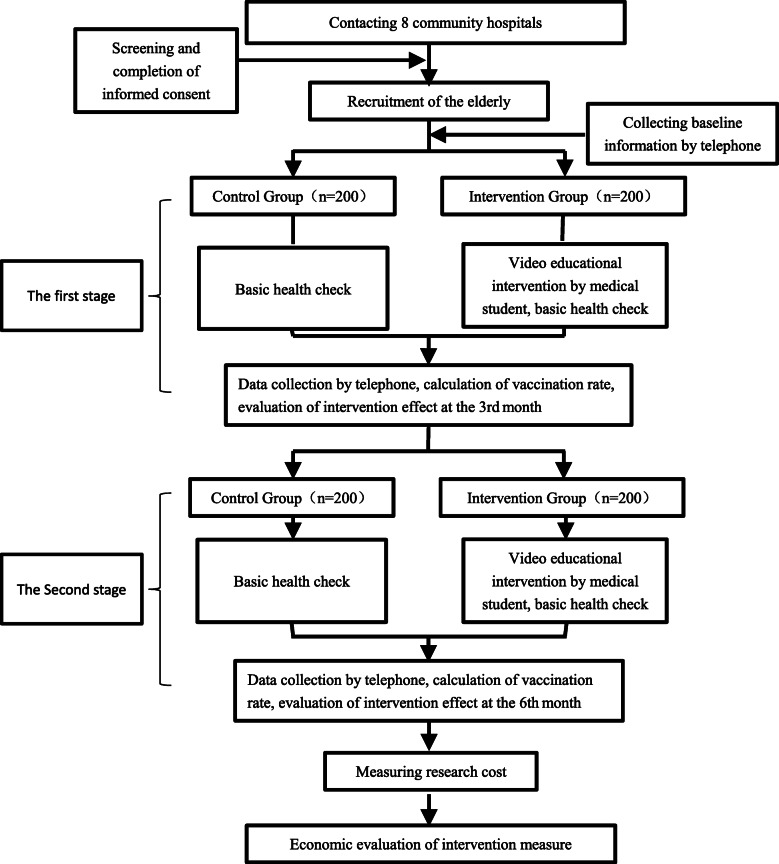
Process of intervention

Formal intervention contains two phases. The first to the third month is the first phase, and the fourth to the sixth month is the second phase. During the first to the second month, the educational intervention and basic health check will be provided to the intervention group in the community hospitals. Initially, a basic health check will first be conducted for participants by medical students. Next, they will be taken to a comfortable room, where a 12 min video will be broadcasted. Then, medical students will answer older adults regarding questions or doubts about the video or influenza vaccine in the next 15 min through a small discussion group. One medical student will be responsible for educating 5 older adults, making them understand the video’s content fully. To guarantee that the older adults actively participate in the discussion, a small gift like a toothbrush will be prepared for them. In contrast, only basic health checks in the control group will be done. In the third month, medical students will make a telephonic call to all enrolled participants to fill in the questionnaire. Then, from the fourth to the sixth month, the procedure will continue as described previously (Fig. 1). After the intervention, effectiveness will be assessed by information collected.

### Outcome indicators

The primary outcomes are willingness to accept influenza vaccination and vaccination coverage, which will be assessed through their self-report. The secondary outcomes are knowledge, attitude, and behavior scores.

### Randomization

All community hospitals in the four districts will be numbered. Then using computer-generated random numbers, community hospitals will be chosen at random. We will use the community health records management system of Xi’an city to export older adults’ health information of community hospitals and order from 1 to n. Corresponding older people through random number software generating 400 numbers will be selected as a sample. After the older adults are recruited, medical students will choose eligible participants and complete informed consent. Using the knowledge, attitudes, and practices (KAP) questionnaire, baseline information will be collected. On account of balancing the baseline, the clusters will be grouped in the intervention and control group randomly through randomization design and Chi-square test.

### Data analysis

Epidata 3.1 will be employed to input data with the help of two investigators. All data collected will be stored on a password-protected computer. Descriptive statistics will be used to present the sociodemographic characteristics of the participants as mean ± standard deviation (M ± SD) and percentage. The chi-square test will be used to compare the difference between vaccination uptake and the correct knowledge rate between the intervention group and the control group before and after the intervention. The t-test will be employed to check the difference in scores of knowledge, attitude, and behavior. A significance level of 5% will be selected in every estimate. The SPSS version 21.0 will be used to perform all analyses.

### Economic evaluation

The economic evaluation will be performed, referring to WHO’s cost-effectiveness and strategic guideline (WHO-CHOICE) [38, 39]. Compared with no intervention, the incremental cost will consist of program costs and intervention costs [39]. The expense of project administration, including connecting with community hospitals, the older adults, and medical students’ salary, will be considered an incremental cost in this study. Incremental health effects deprive of the improvement of uptake of influenza vaccine. The incremental cost-effectiveness ratio will be calculated to judge whether it is economical.

### Ethical statement

Xi’an Jiaotong University’s Research Ethics Committee has approved this study (No. 2020–1183). The written informed consent of the participants will also be obtained during the investigation.

## Discussion

To increase the influenza vaccination rate of older adults, our study will offer 12 min of video intervention and 15 min of group discussion with older adults on the management of medical students. A self-designed KAP questionnaire will be used to assess the difference between knowledge, attitudes, and practice between the intervention group and the control group. The exploratory study could give reference to devise the influenza immunization strategy. To our knowledge, this intervention method combining educational video, medical students with a small group discussion was not used in previous research.

A few intervention studies improving influenza vaccination uptake in older adults were conducted in the Chinese mainland. One study conducted in Ningbo of China [40] showed an increase in the uptake rate and a decrease in the outpatient of upper respiratory tract infection (URI) through comprehensive intervention measures, which included improving the knowledge of the physician, nurses, and older adults, establishing a temporary vaccination point and improving relevant policies. Recommendations by health workers and increasing vaccination sites were the intervention measures employed in the study in Ningbo [41], which revealed that it could promote vaccine coverage.

In the public health system, clinical practitioners often remain busy due to facing the enormous demands of patients [42], so they do not have enough time to educate them. From an economic perspective, the cost of clinic promotion intervention is high, as indicated in a systematic review [43]. Medical students have more free time compared with physicians, nurses, and pharmacists. Every summer or winter vacation Chinese authority requires undergraduates, including medical students, to conduct research or spread scientific knowledge to the public [44]. The video could also be standardized, so it is possible to implement this intervention in a larger area in the future with the cooperation of the health system.

Those medical students who had a 3 min face-to-face education with older people with pamphlets gained a significant increase in the influenza vaccination rate in the Hong Kong study [26]. In our study, the video could be comprehended easily by older adults, as noted in a previous study [25], and 15 min discussion will help them grasp knowledge and reduce their vaccine hesitancy.

With the development of socioeconomic status, older adults pay more attention to their quality of life. It was mentioned to build a lifelong learning system for older people in China’s strategy planning toward the aging problem in 2019. Senior Citizens’ Universities (SCUs) could be a carrier to spread scientific knowledge [45]. In the future, our study will expand on how to conduct health education about influenza in SCUs.

Vaccination policy [31], mostly free vaccination toward influenza [46], can influence the rate according to some studies. Currently, people still need to pay for the influenza vaccine out of pocket in most of the places in China [27, 46]. The government could list it as one of the free vaccines to promote the uptake rate in the future.

Our study has several strengths. First, this study uses medical students to intervene that is cost-effective and can enhance educational effectiveness. Second, the intervention approach of our study is suitable for older adults (urban and rural) due to a video being easy to understand. Third, our study will act as a reference for policymakers to implement influenza vaccination for health authorities. However, there are some limitations to this study. First, the trial will only last 6 months, which can improve older adults’ knowledge to some extent, but the time may not be enough to change the attitude and behavior of older adults. Second, the study’s generalizability will be limited as the study will be undertaken in one city due to limited time, response burden, and inadequate funding. However, this study could be conducted on a large scale if our intervention gets successful results.

## Supplementary Information

**Additional file 1.**

## Data Availability

The datasets generated and analyzed during the current study will be available from the corresponding author on a reasonable request once this trial is finished.

## Abbreviations

KAP: Knowledge, Attitudes, and Practices
HBM: Health Belief Model
ILI: Influenza-like Illness
URTIs: Upper Respiratory Tract Infections
CDC: Centers for Disease Control and Prevention
