# Supplementary material for: Impact of video-led educational intervention on the uptake of influenza vaccine among adults aged 60 years and above in China: a study protocol for a randomized controlled trial
Source: BMC Public Health. 2021 Jan 27;21:222. doi: 10.1186/s12889-021-10220-1 (PMC7839176; doi:10.1186/s12889-021-10220-1)
Supplement: Supplementary file 1 — Additional file 1. [file 12889_2021_10220_MOESM1_ESM.docx]

**Knowledge, attitude, and practice towards influenza and influenza vaccine among the elderly**

**Basic information**

1. Gender: □Male □Female

2. Age (years): □60-69 □70-79 □>80

3. Occupation: □Retirement □Full-time job □Part-time job □others______

4. Education: □Out of school □Primary school □Secondary school

□College school and above

5. Monthly income (RMB): □<1000 □1000-1999 □2000-2999 □3000-3999

□4000-4999 □≥5000

6. Chronic disease: □None □One and above

**Knowledge**

1. There is a difference between the flu and cold.

□Yes □No □Unclear

2. Influenza is a respiratory disease caused by the influenza virus.

□Yes □No □Unclear

3. Influenza is infectious.

□Yes □No □Unclear

4. Influenza is spread mainly by droplets of respiratory secretions.

□Yes □No □Unclear

5. Influenza will bring some complications, such as pneumonia, otitis media.

□Yes □No □Unclear

6. Influenza is mainly divided into type A and type B.

□Yes □No □Unclear

7. Influenza vaccine can prevent influenza effectively.

□Yes □No □Unclear

8. Influenza vaccine needs to be vaccinated annually.

□Yes □No □Unclear

9. The elderly above sixty years old is one of the groups who should receive the influenza vaccine preferentially.

□Yes □No □Unclear

10. Influenza vaccine is divided into trivalent and the quadrivalent influenza vaccine.

□Yes □No □Unclear

**Attitude**

1. The elderly are at higher risk than a common adult of being attacked by flu.

□Strongly agree □Agree □Uncertain □Disagree □Strongly disagree

2. There is a severe disease and economic burden when the elderly have influenza.

□Strongly agree □Agree □Uncertain □Disagree □Strongly disagree

3. Influenza can be treated by antibiotics like amoxicillin and cephalosporins.

□Strongly agree □Agree □Uncertain □Disagree □Strongly disagree

4. There is no need to receive an influenza vaccine for the elderly.

□Strongly agree □Agree □Uncertain □Disagree □Strongly disagree

5. Influenza vaccine is safe.

□Strongly agree □Agree □Uncertain □Disagree □Strongly disagree

6. Influenza won’t occur after receiving the influenza vaccine.

□Strongly agree □Agree □Uncertain □Disagree □Strongly disagree

7. Influenza vaccination should be free.

□Strongly agree □Agree □Uncertain □Disagree □Strongly disagree

8. The community should strengthen education and knowledge propaganda about the prevention of influenza.

□Strongly agree □Agree □Uncertain □Disagree □Strongly disagree

**Practice**

1. Do you take measures to prevent influenza during flu season (such as indoor ventilation, wearing a mask when you go out, washing your hands frequently) ？

□Yes □No

2. Do you strength physical exercises during flu season？

□Yes □No

3. Will you take part in it if the community holds an educational lecture about influenza?

□Yes □No_______

4. If you have an illness like influenza, you will go for self-medication:

□Yes □No

5. Have you received the influenza vaccine recently?

□Yes □No

6. Will you consider influenza vaccination in the future？

□Yes □No
